# Expression Level of Small Envelope Protein in Addition to Sequence Divergence inside Its Major Hydrophilic Region Contributes to More Efficient Surface Antigen Secretion by Hepatitis B Virus Subgenotype D2 than Subgenotype A2

**DOI:** 10.3390/v12090967

**Published:** 2020-09-01

**Authors:** Qianru Wang, Shuwen Fu, Jing Zhang, Quan Yuan, Jisu Li, Ningshao Xia, Yu-Mei Wen, Yongxiang Wang, Shuping Tong

**Affiliations:** 1 Department of Pathobiology, Key Laboratory of Medical Molecular Virology, School of Basic Medical Sciences, Fudan University, Shanghai 200032, China; 17211010049@fudan.edu.cn (Q.W.); fsw_1992@163.com (S.F.); jing_zhang16@fudan.edu.cn (J.Z.); ymwen@shmu.edu.cn (Y.-M.W.); yxwang712@yahoo.com (Y.W.); 2 State Key Laboratory of Molecular Vaccinology and Molecular Diagnostics, School of Public Health, Xiamen University, Xiamen 361102 China; yuanquan@xmu.edu.cn (Q.Y.); nsxia@xmu.edu.cn (N.X.); 3 Liver Research Center, Rhode Island Hospital and Warren Alpert Medical School of Brown University, Providence, RI 02903, USA; ji_su_li_md@brown.edu

**Keywords:** hepatitis B virus, hepatitis B surface antigen, small envelope protein, large envelope protein, genotype, mutation, secretion

## Abstract

Hepatitis B surface antigen (HBsAg) promotes persistent hepatitis B virus (HBV) infection. It primarily corresponds to small (S) envelope protein secreted as subviral particles. We previously found that genotype D clones expressed less S protein than genotype A clones but showed higher extracellular/intracellular ratio of HBsAg suggesting more efficient secretion. The current study aimed to characterize the underlying mechanism(s) by comparing a subgenotype A2 clone (geno5.4) with a subgenotype D2 clone (geno1.2). Five types of full-length or subgenomic constructs were transfected to Huh7 cells at different dosage. HBsAg was quantified by enzyme linked immunosorbent assay while envelope proteins were detected by Western blot. We found that ratio of extracellular/intracellular HBsAg decreased at increasing amounts of DNA transfected. Conflicting findings from two types of subgenomic construct confirmed stronger secretion inhibitory effect of the genotype D-derived large envelope protein. Chimeric constructs followed by site-directed mutagenesis revealed geno1.2 specific V118/T127 and F161/A168 in the S protein as promoting and inhibitory of HBsAg secretion, respectively. In conclusion, more efficient HBsAg secretion by subgenotype D2 than subgenotype A2 is attributed to lower level of S protein expression in addition to V118 and T127 in S protein, although its F161 and A168 sequences rather reduce HBsAg secretion.

## 1. Introduction

Chronic infection with hepatitis B virus (HBV) is a leading cause of liver cirrhosis and hepatocellular carcinoma. According to divergence of their genomic sequences, HBV isolates worldwide can be classified into genotypes A–J. Genotypes B and C predominate in East Asia and are often transmitted vertically from infected mothers. Genotypes A and D co-circulate in many other parts of the world and are transmitted mostly in adulthood through sex, blood transfusion, or shared needles. Serological markers of ongoing HBV infection include hepatitis B e antigen (HBeAg) and hepatitis B surface antigen (HBsAg). HBeAg is a secreted soluble version of core antigen, the building block of core particle (capsid) that shields the viral genome. It was initially identified as the “Australia antigen” [1]. HBsAg is present on the surface of 22 nm subviral particles (SVPs) as well as 42 nm virions. Since SVPs exceed virions by 1000–100,000-fold [2], HBsAg is virtually equivalent to SVPs. SVPs lack capsids inside and are hence genome-free and non-infectious. They promote persistent HBV infection by induction of immune tolerance.

HBsAg is routinely detected from patient sera by its antibody through enzyme linked immunosorbent assay (ELISA). Molecular cloning of the 3.2 kb HBV genome not only identified the S gene as the coding sequence, but also revealed extended coding capacity further upstream. Subsequent experiments in cell culture established that besides major surface protein, also called small (S) envelope protein, HBV expresses large (L) and middle (M) envelope proteins through translation initiation from two upstream in-frame ATG codons [3,4]. That sequence is called preS and subdivided into preS1 and preS2 by these two ATG codons. While S protein consists of the S domain of 226 residues, M protein has N-terminal extension of 55 residues (the preS2 domain). L protein has a further extension of the preS1 domain (119 residues for most genotypes but 108 residues for genotype D). Translation of the three envelope proteins is made possible by transcription of two subsets of co-terminal mRNAs. The 2.4 kb RNA has the entire 1.2 kb envelope gene (preS1/preS2/S) covered at its 5′ end and is used for translation of L protein. The more abundant 2.1 kb RNA has heterogeneous transcription start sites and can express either M or S protein. Transcription of the 2.4 kb and 2.1 kb RNAs is driven by the SPI and SPII promoters, respectively. The SPII promoter overlaps with the preS region.

Expression of S protein alone is sufficient to drive SVP secretion in cell culture, although during natural HBV infection the M protein is also incorporated into SVPs. L protein expressed alone is retained inside cells, and its co-expression with S protein can inhibit SVP secretion to variable extents depending on the L/S protein ratio [5,6,7]. Virion formation (envelopment of core particles) is initiated by L protein interaction with the core protein, while virion secretion is driven by the S protein. The M protein is not essential for the secretion of either SVPs or virions [8,9,10]. Secreted SVPs can promote immune tolerance, whereas intracellular envelope proteins are engaged in virion morphogenesis but may also trigger an immune attack. Thus, the balance between SVP secretion and envelope protein retention as defined by the ratio of extracellular HBsAg/intracellular HBsAg is an important biological trait. In a previous study, we compared envelope protein expression and HBsAg secretion between six genotype A clones and seven genotype D clones derived from patient serum samples [11]. Transient transfection of the human hepatoma cell line Huh7 with dimeric HBV DNA construct (capable of genome replication and expression of all viral proteins) revealed a weaker SPII promoter for genotype D, thus diminishing S protein expression and HBsAg secretion. However, the intracellular levels of HBsAg (by ELISA) and S protein (by Western blot) were even lower, leading to higher ratio of extracellular/intracellular HBsAg by genotype D. Further analysis suggested higher secretion efficiency of the S protein of genotype D than genotype A, which was somewhat offset by stronger inhibitory effect of its L protein [11]. In the present study, we employed one clone each of genotype A and genotype D to characterize the impact of the type of HBV construct and amount of plasmid DNA transfected on the efficiency of HBsAg secretion. In addition, chimeric constructs and site-directed mutants of the 0.7 mer S protein expression construct were employed to identify divergent residues in the 226-aa S protein modulating HBsAg secretion.

## 2. Materials and Methods

### 2.1. DNA Constructs

Replication competent SphI dimer of HBV clone geno5.4 (GenBank accession number: KX827293) of subgenotype A2 and geno1.2 (KX827290) of subgenotype D2 have been described [11]. Replication competent 1.1 mer construct of these two clones was generated by inserting nucleotide sequence 1804–3221/1–1932 of geno5.4 and 1804–3182/1–1932 of geno1.2 into the SacI and HindIII sites of the pcDNA3.1zeo (−) vector in two cloning steps. The 0.7 mer L/M/S construct had genomic positions 2721–3221/1–835 of genotype A and 2715–3182/1–835 of genotype D inserted between SacI–HindIII sites of pBluescript vector, followed by post-transcriptional regulatory element (positions 970–1970) plus SV40 polyA signal inserted between HindIII and XhoI sites. It could express all the three envelope proteins under endogenous HBV promoters and enhancers but not any other HBV proteins [11]. The 0.7 mer S construct was derived from 0.7 mer L/M/S construct by converting the preS1 and preS2 start codons into ACGs: T2855C/T3212C for geno5.4, and T2849C/T3173C for geno1.2. Furthermore, the extra in-frame ATG codon inside geno5.4 was converted to ACG by T2888C mutation. In additional constructs, the S region (nucleotide positions 155–835) was swapped between 0.7 mer S construct of geno5.4 and geno1.2. The CMV-S construct has been described [11]. It has the S gene (genomic positions 155–835) inserted between the EcoRI–HindIII sites of pcDNA3.1/zeo(−) vector leading to S protein expression driven by the CMV promoter. Plasmid DNA was extracted by the plasmid midi kit (Macherey-Nagel, Duren, Germany), followed by phenol and chloroform extraction.

### 2.2. Transient Transfection

The human hepatoma cell line Huh7 was grown in Dulbecco’s Modified Eagle’s Medium supplemented with 10% fetal bovine serum (GIBCO, Mulgrave, Australia). Transient transfection was performed on cells seeded overnight in 6-well plates using Lipofectamine 3000 reagent (Invitrogen, Carlsbad, CA, USA), with pBluescript SKII(−) DNA to bring the total amount of DNA to 2 μg/well. The medium was replaced 12 h later. Both cells and culture supernatant were harvested at day 4 post-transfection. Cells were scraped off and lysed in 100 μL of lysis buffer (10 mM HEPES (pH 7.5), 100 mM NaCl, 1 mM EDTA, and 1% NP-40).

### 2.3. Western Blot Analysis of Secreted and Intracellular Envelope Proteins

A 1/10th volume of the cell lysate was separated by SDS-polyacrylamide gel electrophoresis (PAGE) and transferred to polyvinylidene fluoride membranes. The blots were blocked at room temperature for 1 h with 5% non-fat milk dissolved in TBS–0.1% Tween 20 (TBST). Next, they were incubated at 4 °C overnight with a 1:3000 dilution of polyclonal rabbit anti-HBs antibody (Novus, Centennial, CO, USA) or a 1:5000 dilution of mouse monoclonal anti-preS1 antibody (7H11 [12]) in 5% milk–TBST. Blots were subsequently washed three times (10 min each) with TBST before incubation at room temperature for 1 h with horseradish peroxidase (HRP)-conjugated goat anti-rabbit antibody at 1:10,000 dilution or goat anti-mouse antibody at 1:8000 dilution. The blots were washed again and processed with an enhanced chemiluminescence (Perkin–Elmer, Boston, MA, USA) reagent and exposed to chemiluminescent imaging system (Tanon). For loading control, blots were incubated with a 1:5000 dilution of mouse anti-actin antibody (Proteintech, Chicago, IL, USA; or BBI, Shanghai, China), or a 1:5000 dilution of mouse anti-GAPDH antibody (Proteintech or BBI), followed by a 1:8000 dilution of HRP-conjugated goat anti-mouse antibody in 5% milk–TBST. To detect envelope proteins secreted to culture supernatant by Western blot, 100 or 200 µL of culture supernatant was mixed with 38 or 75 µL of 36% polyethylene glycol 8000 (dissolved in PBS) and rotated at 4 °C overnight. The samples were centrifuged at 12,000× *g* and 4 °C for 1 h and the pellet was resuspended in 10 µL of TN buffer (10 mM Tris-HCl (pH 8.0), 150 mM NaCl), followed by separation of proteins in 0.1% SDS–12.5% polyacrylamide gel (SDS-PAGE). In some experiments, cell lysate or culture supernatant corresponding to same OD_450_ values of HBsAg was used for Western blot analysis (with prior PEG precipitation for culture supernatant).

### 2.4. HBsAg Detection

HBsAg in culture supernatant and cell lysate was detected by a commercial ELISA kit (KHB, Shanghai, China). The samples were properly diluted so that the measured optical density (OD_450_) values of the samples were between 1 and 2, which made the HBsAg concentration in the sample proportional to the optical density value in a linear fashion. Subsequently, the total OD_450_ values of culture supernatant and cell lysate were calculated, based on the dilution and sample volume.

### 2.5. Statistical Analysis

All experiments were repeated three times, and the data were expressed as mean ± SD. GraphPad Prism software version 6.0 was used for statistical analyses. Statistical analyses were performed by one-way ANOVA, and letters show significant differences at *p* < 0.05.

## 3. Results

### 3.1. More Efficient HBsAg Secretion by SphI Dimer of the Genotype D Clone than Genotype A Clone but Reduced Secretion Efficiency at Increasing Amount of DNA Transfected

**Figure 1:**
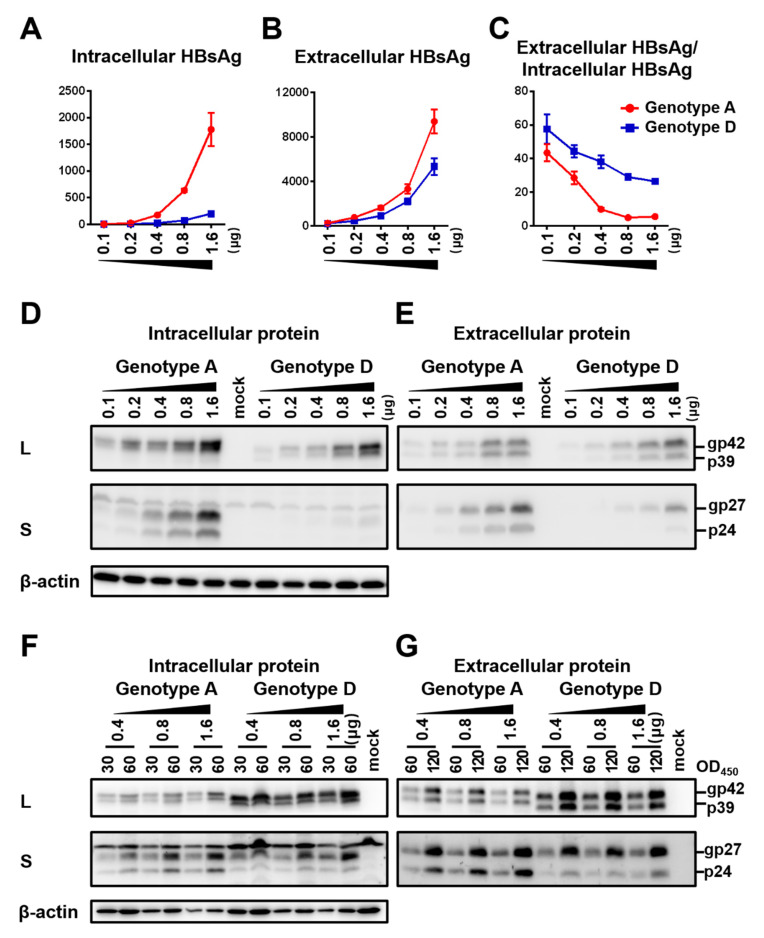
Impact of DNA dosage and viral genotype on envelope protein expression and HBsAg secretion from SphI dimer. Huh7 cells seeded in 6-well plates were transfected with indicated amount of dimer construct of the genotype A clone (geno5.4) or genotype D clone (geno1.2), with the total amount of DNA adjusted to 2 μg/well using pBluescript SKII (−) DNA. Cells and culture supernatant were harvested four days later. (**A**,**B**) HBsAg was measured from cell lysate (**A**) or culture supernatant (**B**) following proper sample dilution to avoid signal saturation, and the total amount of HBsAg (OD_450_) was calculated. Data were averaged from three independent transfection experiments. (**C**) Calculated ratio of extracellular HBsAg/intracellular HBsAg. (**D**,**E**) Western blot analysis of intracellular (**D**) and secreted (**E**) L and S proteins from same amount of cell lysate or culture supernatant. A 1/10th volume of the cell lysate and PEG precipitated extracellular proteins from 200 μL of culture supernatant were separated in SDS-PAGE. Following transfer, the blots were probed with anti-preS1 antibody and anti-S antibody, respectively to reveal L and S proteins. β-actin was used as an intracellular loading control. (**F**,**G**) Western blot analysis of intracellular (**F**) and secreted (**G**) L and S proteins from same amounts of HBsAg. Cell lysate corresponding to 30 or 60 OD_450_ values of HBsAg and culture supernatant corresponding to 60 or 120 OD_450_ values were loaded to protein gels followed by Western blotting with anti-preS1 and anti-HBs antibodies, respectively. β-actin served as intracellular loading control.

The SphI dimer of HBV has two 3.2 kb copies of the HBV genome cloned to pUC18 vector in the same direction. It can transcribe all the HBV RNAs leading to genome replication, envelope protein expression, and HBsAg secretion. Huh7 cells seeded in 6-well plates were transfected with 0.1–1.6 μg of SphI dimer of geno5.4 (genotype A, subgenotype A2) or geno1.2 (genotype D, subgenotype D2). Cells and culture supernatant were harvested four days later. ELISA confirmed dose-dependent increase of HBsAg titers in both cell lysate and culture supernatant (Figure 1A,B). However, the ratio of extracellular/intracellular HBsAg rather decreased at higher DNA dose (Figure 1C). At the highest dose of 1.6 μg geno1.2 produced < 1/10th of intracellular HBsAg than geno5.4, but about half amount of extracellular HBsAg, leading to much higher ratio of extracellular/intracellular HBsAg than geno5.4. Western blot analysis using a polyclonal anti-S antibody revealed less S protein from geno1.2 than geno5.4, with the difference being more striking in cell lysate (Figure 1D,E, second panel). On the other hand, both the intracellular and extracellular levels of L protein were comparable between the two genotypes across different DNA dosage (Figure 1D,E, top panel). To rule out the possibility that higher HBsAg secretion efficiency by geno1.2 or at lower DNA dosage was an artifact of ELISA, we loaded same OD_450_ values of HBsAg into the SDS-PAGE. That generated similar intensity of S protein for the two clones in both cell lysate and culture supernatant when the same DNA dosage was compared, although geno1.2 had higher level of L protein (Figure 1F,G). The same OD values of HBsAg from 0.4 μg of DNA generated comparable intensity of S protein in Western blot as from 1.6 μg of DNA in culture supernatant (Figure 1G), but less signal from cell lysate (Figure 1F). Thus, ELISA rather underestimated the effect of increased DNA dosage at reducing HBsAg secretion.

### 3.2. The 0.7 mer L/M/S Construct Produced Much More L Protein than SphI Dimer, Leading to S Protein Retention and Reversal of Genotypic Difference in Efficiency of HBsAg Secretion

**Figure 2:**
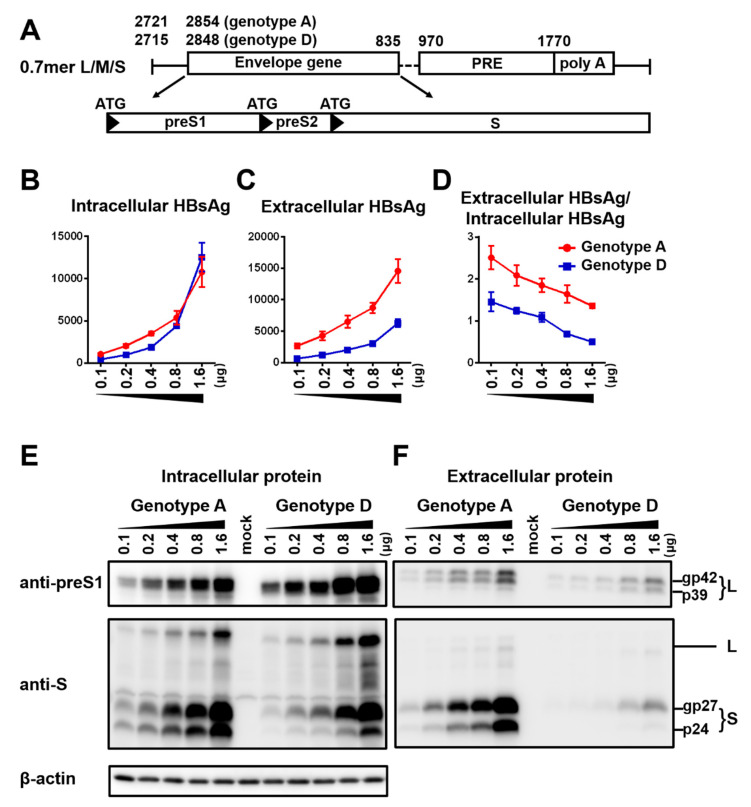
Impact of DNA dosage and viral genotype on envelope protein expression and HBsAg secretion from 0.7 mer L/M/S construct. (**A**) Schematic representation of the 0.7 mer L/M/S construct, with three in-frame ATGs driving L, M, and S protein translation. (**B**–**F**) Huh7 cells seeded in 6-well plates were transfected with indicated amount of 0.7 mer L/M/S construct of geno5.4 (genotype A) or geno1.2 (genotype D), with the total DNA amount adjusted to 2 μg/well. Cells and culture supernatant were harvested four days later. (**B**,**C**) Total amount of HBsAg (OD_450_) from cell lysate (**B**) and culture supernatant (**C**), with data averaged from three independent transfection experiments. (**D**) Calculated ratio of extracellular/intracellular HBsAg. (**E**,**F**) Western blot analysis of intracellular (**E**) and extracellular (**F**) L and S proteins from same volume of cell lysate or culture supernatant by anti-preS1 and anti-HBs antibodies, respectively. β-actin served as an intracellular loading control.

**Figure 3:**
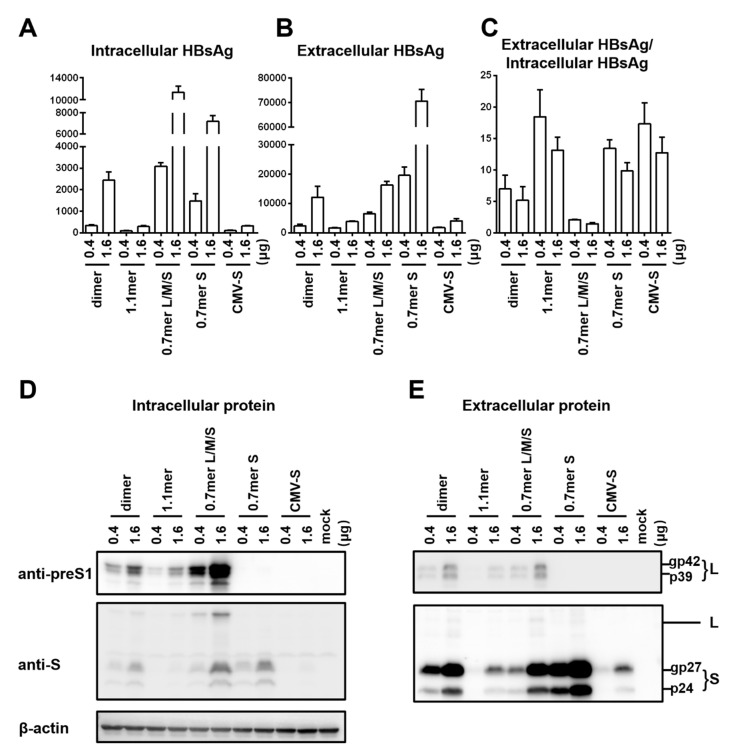
Comparison of five types of replication or expression constructs for efficiency of envelope protein expression and HBsAg secretion: the genotype A clone. Huh7 cells in 6-well plates were transfected with 0.4 or 1.6 μg of various HBV DNA constructs of geno5.4 (genotype A). Cells and culture supernatant were harvested four days later. (**A**,**B**) Total amount of HBsAg (OD_450_) from cell lysate (**A**) and culture supernatant (**B**). Data were averaged from three independent transfection experiments. (**C**) The calculated ratio of extracellular/intracellular HBsAg. (**D**,**E**) Western blot analysis of intracellular (**D**) and extracellular (**E**) L and S proteins from same volume of cell lysate or culture supernatant by anti-preS1 and anti-HBs antibodies, respectively. β-actin was used as an intracellular loading control.

**Figure 4:**
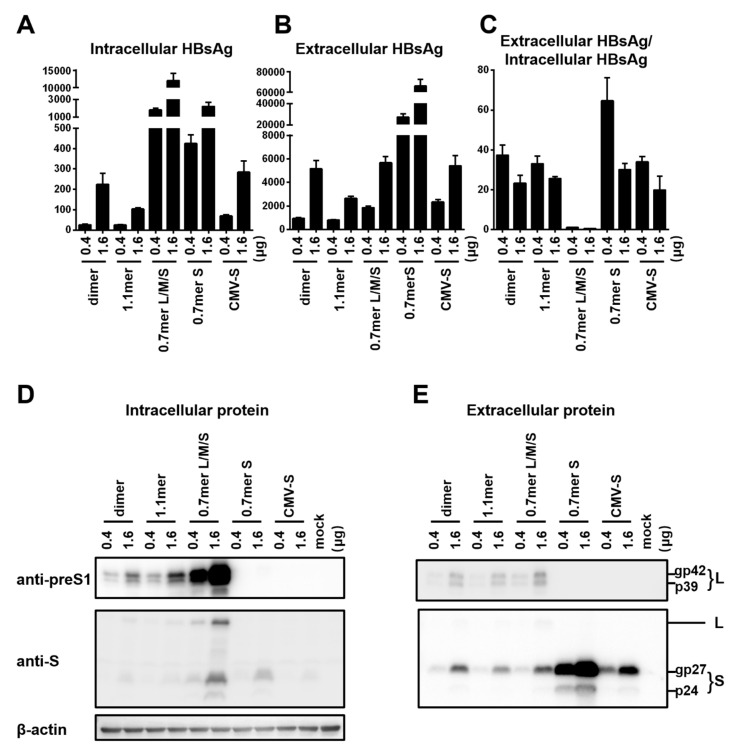
Comparison of five types of replication or expression constructs for efficiency of envelope protein expression and HBsAg secretion: the genotype D clone. Huh7 cells in 6-well plates were transfected with 0.4 or 1.6 μg of various HBV DNA constructs of geno1.2 (genotype D). Cells and culture supernatant were harvested four days later. (**A**,**B**) Calculated total amount of HBsAg (OD_450_) from cell lysate (**A**) and culture supernatant (**B**), with data averaged from three independent transfection experiments. (**C**) Calculated ratio of extracellular/intracellular HBsAg. (**D**,**E**) Western blot analysis of intracellular (**D**) and extracellular (**E**) L and S proteins from same volume of cell lysate or culture supernatant by anti-preS1 and anti-HBs antibodies, respectively. β-actin served as an intracellular loading control.

To compare HBsAg secretion between the two genotypes without complication of genome replication and other viral proteins, we used a 0.7 mer L/M/S construct for transcription of 2.4 kb and 2.1 kb RNAs under endogenous SPI and SPII promoters. Relative to the SphI dimer, the 0.7 mer L/M/S construct of geno1.2 released much less HBsAg to culture supernatant than geno5.4 (compare Figure 2C with Figure 1B). On the other hand, intracellular level of HBsAg increased to become similar to that of geno5.4, especially at highest DNA dose (Figure 2B vs. Figure 1A). Consequently from 0.7mer L/M/S construct, geno1.2 produced a lower ratio of extracellular/intracellular HBsAg than geno5.4 (Figure 2D). In this regard the 0.7 mer L/M/S construct displayed a much higher intracellular level of HBsAg than SphI dimer for geno5.4 and especially geno1.2 (Figure 3A and Figure 4A), when the extracellular HBsAg levels were similar (Figure 3B and Figure 4B). Consequently the 0.7 mer L/M/S construct had reduced ratio of extracellular/intracellular HBsAg than SphI dimer, which was dramatic for geno1.2 (Figure 3C and Figure 4C). Western blot revealed that the 0.7 mer construct of geno1.2 manifested more intracellular but less secreted L protein than geno5.4 (Figure 2E,F, top panels). Our previous study found the L protein of genotype D was more inhibitory of HBsAg secretion than that of genotype A [11]. In this regard the 0.7 mer L/M/S construct produced much more intracellular L protein than the SphI dimer, especially for geno1.2 (Figure 3D and Figure 4D).

### 3.3. Ablating L/M Protein Expression from the 0.7 mer Construct Markedly Enhanced HBsAg Secretion and Restored More Efficient HBsAg Secretion from the Genotype D Clone, Especially at a Low DNA Dosage

**Figure 5:**
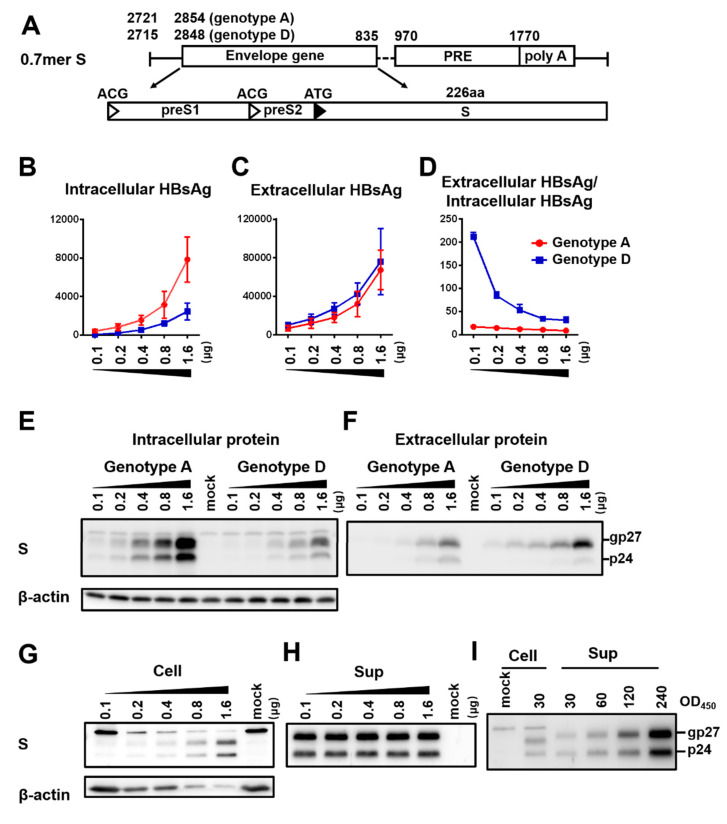
Impact of DNA dosage and viral genotype on S protein expression and HBsAg secretion from 0.7mer S protein construct. (**A**) The 0.7mer S construct was derived from 0.7mer L/M/S construct by mutating the preS1 and preS2 ATG codons into ACG. (**B**–**F**) Huh7 cells in 6-well plates were transfected with indicated amount of 0.7mer S protein construct of genotype A (geno5.4) or genotype D (geno1.2). Cells and culture supernatant were harvested four days later. (**B**,**C**) Total amount of HBsAg (OD_450_) from cell lysate (**B**) and culture supernatant (**C**). Data were averaged from three independent transfection experiments. (**D**) Calculated ratio of extracellular/intracellular HBsAg. (**E**,**F**) Western blot analysis of intracellular (**E**) and extracellular (**F**) S protein from same volume of cell lysate or culture supernatant. β-actin served as an intracellular loading control. (**G**,**H**) Loading with equal OD_450_ values of HBsAg from different doses of 0.7mer S construct of geno5.4 transfected. The OD_450_ values were 30 for cell lysate (**G**) and 300 for culture supernatant (**H**). β-actin served as loading control for cell lysate. (**I**) Comparison of S protein from 30 OD_450_ values of HBsAg from cell lysate with 30–240 OD_450_ values of HBsAg from culture supernatant. Huh7 cells transfected with 1.6 μg of geno5.4 were the source of cell lysate and culture supernatant.

To confirm that the less efficient HBsAg secretion by geno1.2 than geno5.4 from 0.7 mer L/M/S construct in contrast to SphI dimer was attributed to different L/S protein ratios, we prevented L/M protein expression from the 0.7 mer construct by mutating their translation initiation codons (Figure 5A). The resultant 0.7 mer S protein construct of geno1.2 displayed much less intracellular S protein than geno5.4, but similar or more S protein in culture supernatant (Figure 5E,F). ELISA of intracellular and extracellular HBsAg revealed a similar pattern (Figure 5B,C). The ratio of extracellular/intracellular HBsAg was > 10 times higher for geno1.2 than geno5.4 when 0.1 μg of DNA was transfected, although the difference became less striking at higher DNA doses (Figure 5D). To exclude the possibility that higher HBsAg secretion efficiency at lower DNA dosage was an error of ELISA, the same OD_450_ values of S protein from Huh7 cells transfected with different doses of geno5.4 were compared in Western blot. Little difference in S protein (gp27/p24) intensity was found for 300 OD_450_ value of HBsAg from culture supernatant of cells transfected with 0.1–1.6 μg 0.7 mer S construct (Figure 5H). For cell lysate, 30 OD_450_ value of HBsAg from lower DNA doses generated stronger signals of β-actin and a non-specific band in anti-S Western blot (as anticipated) but weaker signals of gp27/p24 (Figure 5G). Therefore, when only S protein was expressed ELISA rather overestimated intracellular HBsAg at lower DNA dosage, thus higher HBsAg secretion efficiency at lower DNA dosage could be confirmed by Western blot analysis.

### 3.4. Both the S Region and a Weaker SPII Promoter Contributed to More Efficient HBsAg Secretion from the 0.7 mer S Construct of geno1.2 than geno5.4

**Figure 6:**
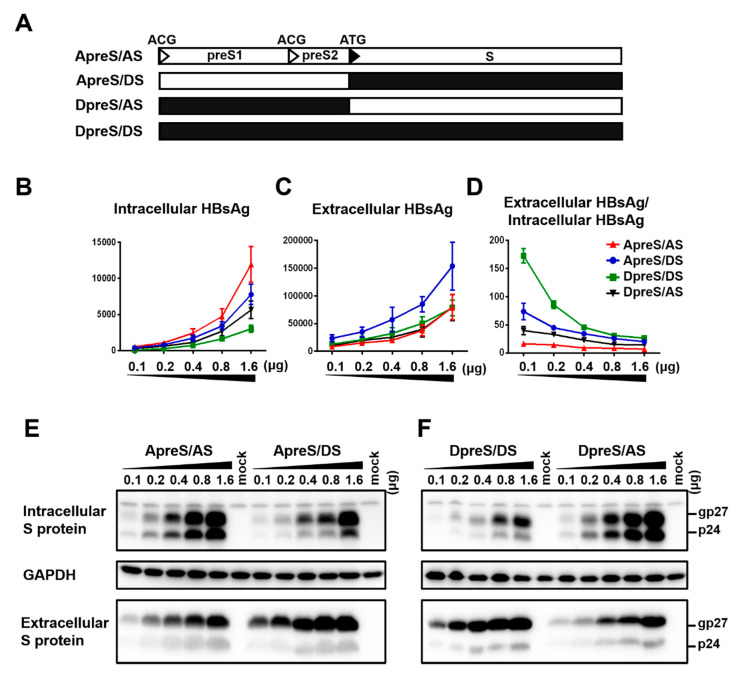
Chimeric 0.7 mer S protein constructs to dissect the relative contribution of the S region vs. upstream sequence on efficiencies of S protein expression and HBsAg secretion. (**A**) Schematic representation of the four types of 0.7 mer S protein construct: original geno5.4 (ApreS/AS) and geno1.2 (DpreS/DS), chimeric ApreS/DS and DpreS/AS. (**B**–**F**) Huh7 cells in 6-well plates were transfected with 0.1–1.6 μg of the four types of 0.7 mer S protein construct. Cells and culture supernatant were harvested four days later. (**B**,**C**) Total amount of HBsAg (OD_450_) from cell lysate (**B**) and culture supernatant (**C**). Data were averaged from three independent transfection experiments. (**D**) Calculated ratio of extracellular/intracellular HBsAg. (**E**) Comparison of ApreS/AS with ApreS/DS for intracellular and extracellular S protein. (**F**) Comparison of DpreS/DS with DpreS/AS for intracellular and extracellular S protein. For both panels, same volume of cell lysate or culture supernatant was used, and GAPDH served as loading control for cell lysate.

From our previously study, reduced S protein expression by the genotype D clones than genotype A clones was attributed to a weaker SPII promoter [11]. Since the current work revealed a reverse correlation between the DNA dosage and ratio of extracellular/intracellular HBsAg, the higher HBsAg secretion efficiency of the 0.7 mer S construct of geno1.2 than geno5.4 could be partly attributed to a lower level of S protein expression. We therefore swapped the S region to generate two chimeric constructs. The original 0.7 mer construct of geno5.4 and geno1.2 (labeled as ApreS/AS and DpreS/DS, respectively) as well as their chimeras (ApreS/DS; DpreS/AS) were transfected in parallel to Huh7 cells. The preS sequence of geno1.2 was associated with lower intracellular and sometimes also extracellular levels of HBsAg, but higher HBsAg secretion efficiency (Figure 6B–D; compare DpreS/AS with ApreS/AS; DpreS/DS with ApreS/DS). When the preS sequence was the same, the S region of geno1.2 was often associated with lower intracellular level but higher extracellular level of HBsAg than that of geno5.4, leading to higher secretion efficiency (Figure 6B–D; compare ApreS/DS with ApreS/AS; DpreS/DS with DpreS/AS). Western blot analysis confirmed overall lower intracellular level but higher extracellular level of S protein in association with the S gene of geno1.2 (Figure 6E,F). Thus, the S protein of geno1.2 still had higher secretion efficiency than that of geno5.4 when S protein expression was driven by the same SPII promoter.

### 3.5. Reduced HBsAg Production by 1.1 mer Construct than SphI Dimer and Much Reduced S Protein Expression by CMV-S Construct than 0.7 mer S Construct

Results presented so far raised concern that the type of HBV DNA construct used to compare HBsAg secretion among clinical isolates could influence the findings due to altered level of S protein expression or changed L/S protein ratio. To substantiate this point, we compared SphI dimer, 0.7 mer L/M/S construct, and 0.7 mer S construct of the same genotype together using just two DNA doses (0.4 μg and 1.6 μg). Also included were CMV-S construct with the S gene alone cloned downstream of the strong CMV promoter, and 1.1 mer construct with transcription of the 3.5 kb pregenomic (pg) RNA is driven by the CMV promoter. The 1.1 mer construct of geno5.4 produced less L protein and even less S protein than corresponding SphI dimer, in both cell lysate and culture supernatant (Figure 3D,E, top and second panels). The HBsAg titers were reduced, with the ratio of extracellular/intracellular HBsAg rather increased (Figure 3A–C). For geno1.2, the 1.1 mer construct showed no reduction of L protein and only limited reduction of S protein (Figure 4D,E). It displayed similar HBsAg secretion efficiency as the SphI dimer despite somewhat reduced levels of both intracellular and extracellular HBsAg (Figure 4A–C). The CMV-S construct produced much less S protein than 0.7 mer S protein construct, in both cell lysate and culture supernatant, especially for geno5.4 (Figure 3D,E and Figure 4D,E). ELISA confirmed much less HBsAg from the CMV-S construct (Figure 3A,B and Figure 4A,B).

### 3.6. Chimeric Constructs within the S Region Identified both Positive and Negative Regulators of HBsAg Secretion from geno1.2

**Figure 7:**
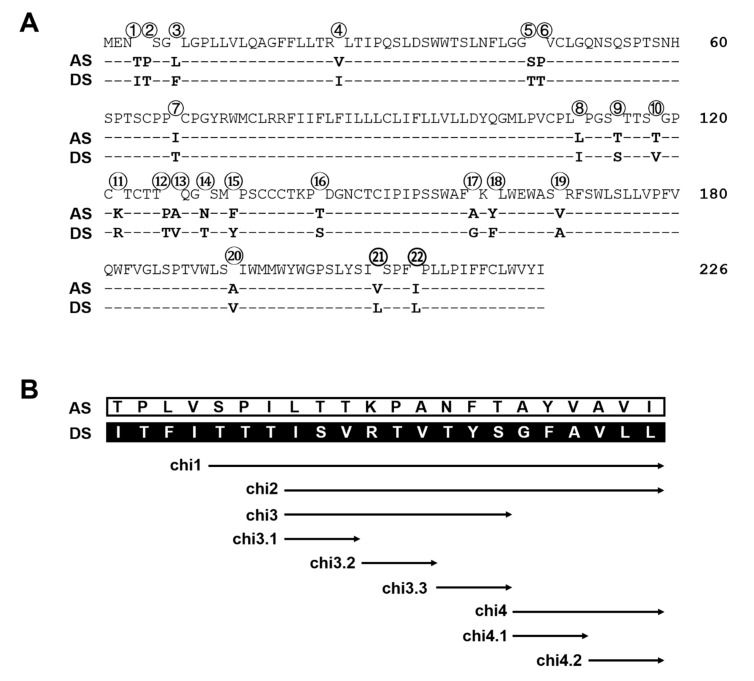
Divergent residues in the S protein between geno5.4 and geno1.2 and chimeric constructs to map the determinants for efficient HBsAg secretion. (**A**) The 22 divergent residues between geno5.4 and geno1.2 on the S protein of 226 residues. (**B**) The nine pairs of chimeric constructs of the S region between geno5.4 and geno1.2.

**Figure 8:**
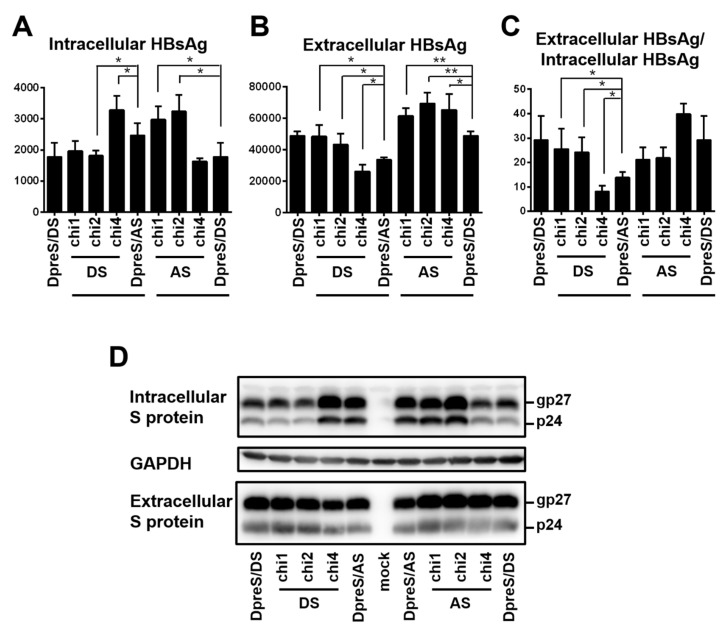
Three pairs of chimeric constructs between DpreS/AS and DpreS/DS to map the determinants for efficient HBsAg secretion. Huh7 cells in 6-well plates were transfected with 0.7 mer S construct of DpreS/AS, DpreS/DS, or three chimeric constructs, followed by HBsAg measurement from both cell lysate and culture supernatant. For the left part of panels A–D, chi1, chi2, and chi4 with DS (lanes 2–4) are DpreS/AS with various parts of the S region replaced with geno1.2, while DpreS/DS (lane 1) had the entire S region replaced with geno1.2. For the right part, chi1, chi2, and chi4 with AS (lanes 6–8) are DpreS/DS with various parts of the S region replaced with geno5.4, while DpreS/AS (lane 5) had the entire S region replaced. (**A**–**C**) Total intracellular (**A**) and extracellular (**B**) levels of HBsAg (OD_450_) averaged from three independent transfection experiments, and the calculated ratio of extracellular/intracellular HBsAg (**C**). Statistical difference between each chimeric construct and its parental construct was analyzed by one-way ANOVA (* *p* < 0.05, ** *p* < 0.01). (**D**) Western blot analysis of intracellular and extracellular S protein from same volume of cell lysate or culture supernatant, with GAPDH serving as loading control for cell lysate.

For the S protein of 226 residues, geno1.2 differed from geno5.4 at 22 positions (Figure 7A). Chimeric constructs of the S region were employed to map the determinants for genotypic difference in HBsAg secretion. The overlapping chimeric constructs chi4, chi2, and chi1 covered 6, 15, and 18 divergent positions from the C-terminus, respectively (Figure 7B). For DpreS/AS, its chi1 and chi2 with the S region of geno1.2 reduced intracellular level of HBsAg but increased extracellular level, leading to higher HBsAg secretion efficiency approaching that of DpreS/DS (Figure 8A–C, compare lanes 2, 3, 5). Surprisingly, chi4 of DpreS/AS showed increased intracellular HBsAg but reduced extracellular HBsAg to further reduce HBsAg secretion (Figure 8A–C, lanes 4, 5). As for DpreS/DS, ratio of extracellular/intracellular HBsAg was reduced in chi1 and chi2 with the S region of geno5.4, but further increased in chi4 (Figure 8C, lanes 6–9). Chimeric constructs chi1, chi2, and chi4 were also made in the background of ApreS/AS and ApreS/DS, and transfection experiments generated similar findings (Figure S1).

### 3.7. Fine Mapping of the Positive and Negative Regulators of HBsAg Secretion from geno1.2

**Figure 9:**
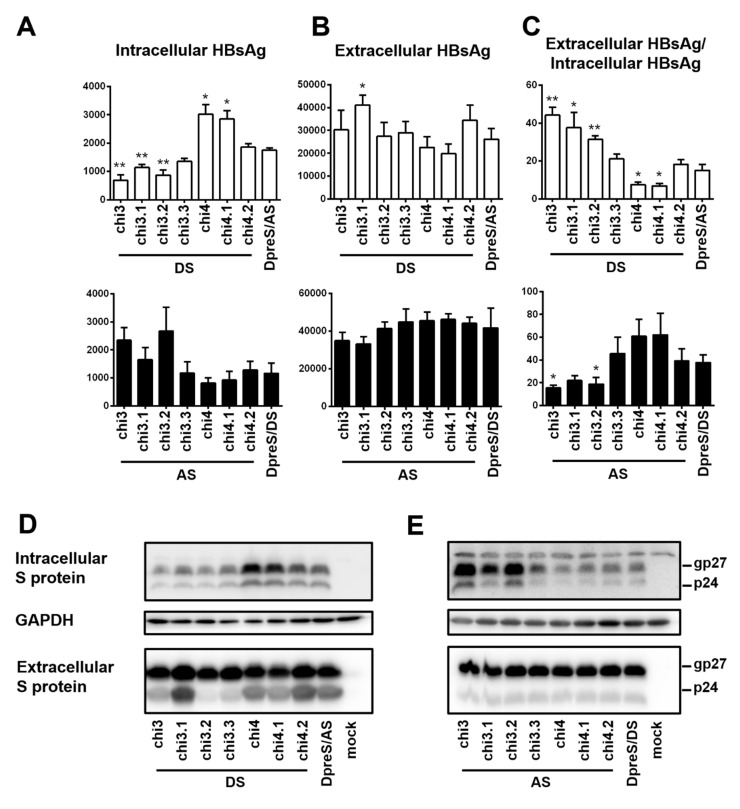
Five pairs of short chimeric constructs between DpreS/AS and DpreS/DS to further narrow down the determinants for efficient HBsAg secretion. Huh7 cells in 6-well plates were transfected with 0.7 mer S construct of DpreS/AS or DpreS/DS, or chimeric construct chi3 or chi4, or even shorter chimeras (chi3.1, chi3.2, chi3.3; chi4.1, chi4.2). (**A**–**C**) Total intracellular (**A**) and extracellular (**B**) levels of HBsAg (OD_450_) averaged from three independent transfection experiments and the calculated ratio of extracellular/intracellular HBsAg (**C**). Statistical difference between the original construct (DpreS/AS or DpreS/DS) and a chimeric construct was analyzed by one-way ANOVA (* *p* < 0.05, ** *p* < 0.01). The top panels are based on DpreS/AS while the lower panels are based on DpreS/DS. (**D**,**E**) Western blot analysis of intracellular and extracellular S protein from the DpreS/AS series (**D**) or DpreS/DS series (**E**). Same volume of cell lysate or culture supernatant was used, and GAPDH served as loading control for cell lysate.

Chi4 covered six divergent residues at the C-terminus of S protein, which were part of 15 divergent residues covered by chi2 (Figure 7B). The opposite effects of chi4 and chi2 on HBsAg secretion (Figure 8) suggested that divergent residues 8–16 were responsible for more efficient HBsAg secretion by geno1.2 than geno5.4 (Figure 7B). A chimeric construct covering only this portion of the S region was generated (chi3). Moreover, three shorter chimeric constructs covering divergent residues 8–10 (chi3.1), 11–13 (chi3.2), and 14–16 (chi3.3) were generated to fine map the responsible sequence (Figure 7B). Based on the measurement of extracellular and intracellular HBsAg, and calculation of their ratio from three independent transfection experiments, chi3.1 and chi3.2 of DpreS/AS increased efficiency of HBsAg secretion, albeit not to the extent of chi3 (Figure 9A–C, top panel). For chimeric constructs based on DpreS/DS, the ratio of extracellular/intracellular HBsAg was reduced in chi3.1 and chi3.2, but not chi3.3 (Figure 9A–C, bottom panel). Therefore, divergent residues 8–13 were largely responsible for more efficient HBsAg secretion by geno1.2 than geno5.4. In terms of the C-terminal determinant of HBsAg secretion, we made chi4.1 and chi4.2 covering divergent residues 17–19 and 20–22, respectively (Figure 7B). For both DpreS/AS- and DpreS/DS-based chimeric constructs, chi4.1 displayed similar ratio of extracellular/intracellular HBsAg as chi4, whereas chi4.2 showed little change from the parental constructs (Figure 9C). Therefore, geno1.2-derived divergent residues 17–19 supported less efficient HBsAg secretion. The same set of chimeric constructs (chi3.1, chi3.2, chi3.3, chi4.1, and chi4.2) were analyzed for both ApreS/AS and ApreS/DS, with similar findings (Figure S2).

### 3.8. HBsAg Secretion Could be Increased by V118 and T127 but Diminished by F161 and A168 in the S Protein of geno1.2

**Figure 10:**
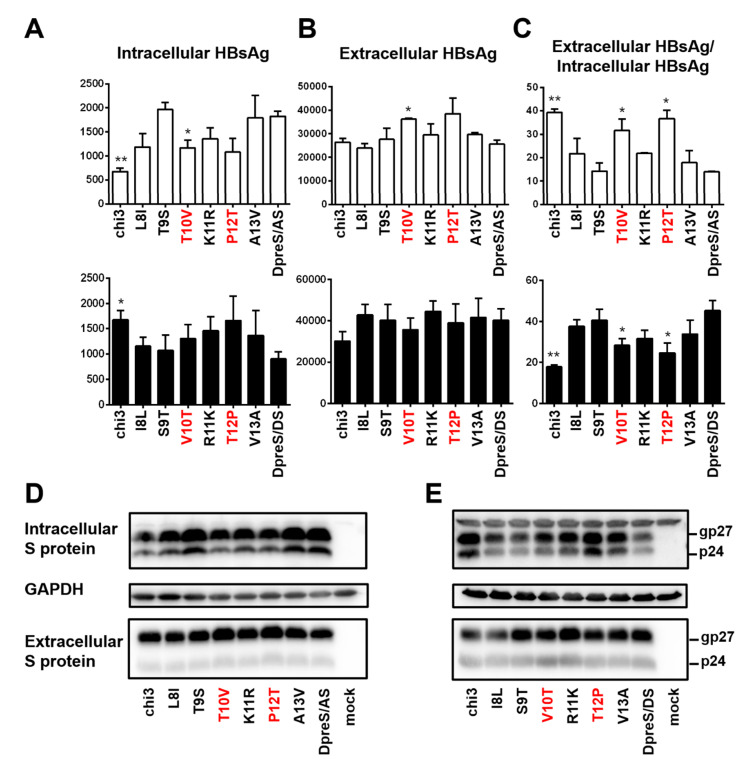
Site-directed mutagenesis to identify residues in the S protein responsible for efficient HBsAg secretion by geno1.2. Huh7 cells in 6-well plates were transfected with 0.7 mer S construct of DpreS/AS or DpreS/DS, or chimeric construct chi3, or one of the six site-directed mutants targeting divergent residues 8–13. (**A**–**C**) Total intracellular (**A**) and extracellular (**B**) levels of HBsAg (OD_450_) averaged from three independent transfection experiments, and the calculated ratio of extracellular/intracellular HBsAg (**C**). The top and bottom panels are based on DpreS/AS and DpreS/DS, respectively. Statistical difference between the original construct (DpreS/AS or DpreS/DS) and a mutant construct was analyzed by one-way ANOVA (* *p* < 0.05, ** *p* < 0.01). (**D**,**E**) Western blot analysis of intracellular and extracellular S protein from the DpreS/AS series (**D**) and DpreS/DS series (**E**). Same volume of cell lysate or culture supernatant was used, and GAPDH served as loading control for cell lysate.

To identify residues responsible for more efficient HBsAg secretion by geno1.2 than geno5.4, divergent residues 8–13 were individually mutated. For residues covered by chi3.1, the T10V substitution of DpreS/AS was more effective at increasing the ratio of extracellular/intracellular HBsAg than L8I, while T9S had little effect (Figure 10C, top panel). Conversely, V10T mutation of DpreS/DS modestly reduced the HBsAg ratio while I8L and especially S9T had little effect (Figure 10C, lower panel). For residues covered by chi3.2, the P12T mutation of DpreS/AS was more effective than K11R or A13V in reducing intracellular HBsAg while increasing extracellular HBsAg to promote HBsAg secretion (Figure 10A–C, top panel). In the reverse approach, the T12P change in DpreS/DS was more effective than R11K or V13A change in retaining HBsAg (Figure 10A–C, lower panel). Since the divergent residues 10 and 12 correspond to positions 118 and 127 in S protein, respectively, these findings suggested that V118 and T127 in the S protein of geno1.2 promoted HBsAg secretion relative to T118 and P127 found in geno5.4.

**Figure 11:**
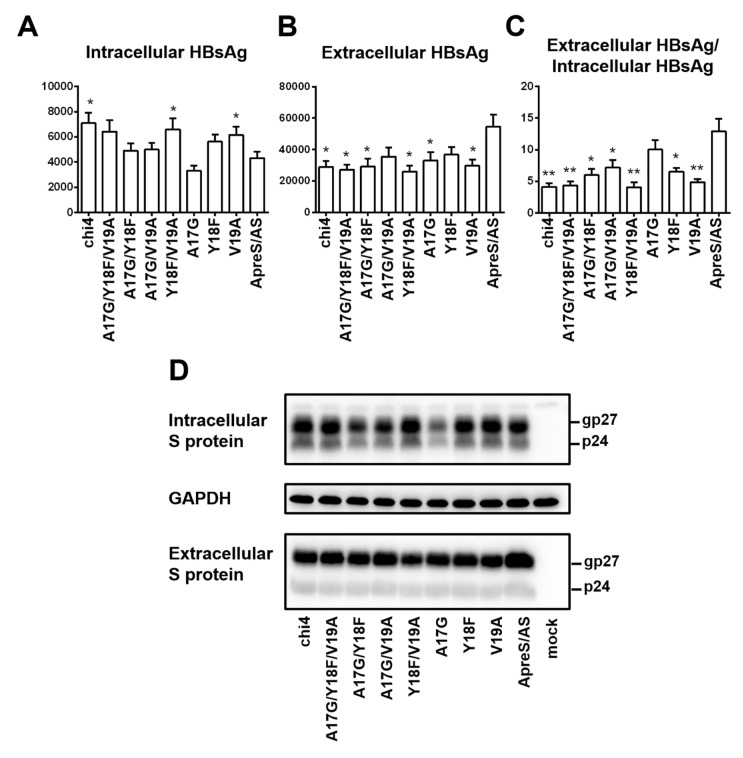
Site-directed mutagenesis to identify residues in the S protein that negatively regulate HBsAg secretion from geno1.2. The geno1.2-specific divergent residues 17, 18, 19, or their various combinations were introduced to the 0.7 mer S construct of geno5.4 (ApreS/AS). The parental construct, chi4, and seven site-directed mutants were transfected to Huh7 cells in 6-well plates. (**A**–**C**) Total intracellular (**A**) and extracellular (**B**) levels of HBsAg (OD_450_) averaged from three independent transfection experiments, and the calculated ratio of extracellular/intracellular HBsAg (**C**). Statistical difference between the original construct (ApreS/AS) and a mutant construct was analyzed by one-way ANOVA (* *p* < 0.05, ** *p* < 0.01). (**D**) Western blot analysis of intracellular and extracellular S protein from same volume of cell lysate or culture supernatant. GAPDH served as loading control for intracellular S protein.

Chi4.1 covered divergent residues 17–19. We introduced single, double, or triple amino acid substitutions into the ApreS/AS construct to identify the geno1.2-specific residues suppressing HBsAg secretion. Of the three single point mutations, V19A and to a lesser extent Y18F increased intracellular HBsAg to reduce the ratio of extracellular/intracellular HBsAg, while A17G had limited effect (Figure 11A–C). Among the three double mutants, only Y18F/V19A markedly increased intracellular HBsAg to reduce HBsAg secretion similar to chi4.1. Since divergent residues 18 and 19 correspond to positions 161 and 168 in the S protein, respectively, our findings suggest that the Y161F and V168A substitutions in the S protein of geno5.4 to mimic geno1.2 could reduce HBsAg secretion.

## 4. Discussion

Secreted HBsAg may promote immune tolerance during virus transmission to a new host, while intracellular HBsAg could serve as an immune target for viral clearance later on. HBsAg seroconversion (loss of HBsAg followed by subsequent rise of anti-HBs antibodies) is the therapeutic end point [13,14]. However, host and viral factors regulating S protein expression and HBsAg secretion remain incompletely understood, including the impact of viral genotype. HBV genotypes A and D show overlapping geographic distributions but differ in their modes of transmission, propensity to induce chronic infection, and response to interferon therapy [15]. A clinical study in Europe found higher mean pre-treatment serum HBsAg titer in genotype A than genotype D samples [16]. By transfecting Huh7 cells with 1.2 mer construct of genotypes A (A1 and A2 subtypes), B (B1 and B2 subtypes), C, and D, Sugiyama et al. found HBsAg production to be highest for subtype A2 and lowest for genotype D [17]. We recently performed transfection experiments of SphI dimer of seven genotype D clones (of D1, D2, D3, or D7 subtype) and 6 genotype A clones (all of the A2 subtype). In both Huh7 and HepG2 cells the genotype D clones produced less HBsAg in culture supernatant, which could be attributed to a weaker SPII promoter activity to diminish transcription of the 2.1 kb RNA [11]. Since the genotype D clones displayed even less HBsAg in cell lysate, they had higher ratio of extracellular/intracellular HBsAg suggesting more efficient HBsAg secretion. Further analysis revealed that the S protein of genotype D had higher intrinsic secretion efficiency, which was partly offset but the stronger inhibitory effect of its L protein [11].

To further characterize more efficient HBsAg secretion by genotype D than genotype A, the present study focused on geno5.4 of the A2 subtype and geno1.2 of the D2 subtype. The two clones were compared in the context of different types of replication (SphI dimer, 1.1 mer) or envelope protein construct (0.7 mer L/M/S, 0.7 mer S, CMV-S), with different DNA dosage. Consistent with a role of the L protein in inhibiting S protein secretion [5,6,7], the 0.7 mer L/M/S construct secreted less HBsAg to culture supernatant while retaining more HBsAg inside cells than the 0.7mer S construct. The difference was much more striking for geno1.2 (Figure 4A–C) than geno5.4 (Figure 3A–C), thus reinforcing a greater inhibitory effect of the genotype D-derived L protein [11]. The 0.7 mer L/M/S construct also produced lower ratio of extracellular/intracellular HBsAg than the SphI dimer (Figure 3C and Figure 4C), in correlation with much higher expression of L protein, especially for geno1.2 (Figure 3D and Figure 4D). That could explain why in contrast to all the other types of constructs, from 0.7 mer L/M/S construct geno1.2 rather manifested lower HBsAg secretion efficiency than geno5.4 (Figure 2D).

We suspect that the 0.7 mer L/M/S construct produces more L protein than corresponding SphI dimer through increased transcription of the 2.4 kb RNA. All the HBV RNAs are unidirectional and co-terminal, thus raising the possibility of transcriptional interference. Our previous work revealed ability of the 3.5 kb pgRNA to diminish transcription of the 2.4 kb, 2.1 kb, and 0.7 kb RNAs from dimer or 1.1 mer construct [18]. With 0.7 mer L/M/S construct transcription unit for the 2.4 kb RNA becomes the 5′-most and no longer subject to inhibition by the 3.5 kb RNA. That may increase transcription of 2.4 kb RNA relative to the 2.1 kb and 0.7 kb RNAs. Similarly, less L and S protein production from the 1.1 mer construct of geno5.4 than its SphI dimer (Figure 3D,E) could be explained by reduced transcription of 2.4 kb and 2.1 kb RNAs secondary to the overproduction of 3.5 kb pgRNA. Certainly, Northern blot or primer extension assay is needed to verify this hypothesis.

An interesting novel finding from the present study was the inverse correlation between DNA dosage and efficiency of HBsAg secretion, which was true for all the five types of construct analyzed, for both geno5.4 and geno1.2 (Figure 1, Figure 2, Figure 3, Figure 4, Figure 5 and Figure 6). For the 0.7 mer S construct the dose effect was more striking for geno1.2 than geno5.4 (Figure 5D). Inspection of Western blot images of intracellular and extracellular S protein did not easily give such an impression, possibly due to the difficulty to quantify very low or very high level of S protein from the Western blot. ELISA is simpler and much more sensitive than Western blot. We managed to achieve OD_450_ value of 1–2 for all samples by different fold of dilution according to the type of construct, DNA dosage, viral genotype, and cell lysate vs. culture supernatant. Moreover, control experiments where same OD_450_ values of HBsAg from cell lysate or culture supernatant were analyzed by Western blot revealed no bias against the S protein of SphI dimer of genotype A or D (Figure 1F,G). ELISA rather overestimated S protein from lysate but not culture supernatant of cells transfected with low dose of SphI dimer or 0.7mer S construct (Figure 1F,G and Figure 5G,H). This might be attributed to the presence of cross-reactive host protein(s) in cell lysate, which will contribute more significantly to “HBsAg” value when S protein expression is reduced at low DNA dose. At least for Western blot, a non-specific band about gp27 could be picked up from cell lysate by the anti-HBs antibody, which became increasingly dominant for the low DNA dose when 30 OD_450_ values of HBsAg were analyzed (Figure 5G). No non-specific band was detectable from culture supernatant (Figure 5I).

The inverse correlation between the DNA dosage (amount of S protein expressed) and HBsAg secretion efficiency could at least partly explain why the 1.1 mer construct of geno5.4 manifested higher secretion efficiency than corresponding SphI dimer (Figure 3A–C), and why genotype D clones showed higher secretion efficiency than genotype A clones [11]. Consistent with a weaker SPII promoter for genotype D clones [11], replacing the preS region of geno1.2 with that of geno5.4 increased both intracellular and extracellular levels of HBsAg (Figure 6B,C). However, ratio of extracellular/intracellular HBsAg rather reduced, although still higher than the pure 0.7 mer S construct of geno5.4 (Figure 6D). Replacing the preS region of geno5.4 with that of geno1.2 produced opposite effects, but again confirming the effect of both expression level and sequence of the S protein on much higher HBsAg secretion efficiency of the 0.7 mer S construct of geno1.2 than geno5.4. SVPs are secreted by constitutive secretory pathway [19,20,21]. Molecular chaperones such as HSc70, calnexin, and BiP are implicated in assembly/morphogenesis of HBV SVPs by interacting with S or L protein [20,22,23,24]. Less efficient HBsAg secretion at higher level of S protein expression might be attributed to saturation of such host co-factors.

Surprisingly, the CMV-S construct produced much less S protein than the 0.7 mer S construct, as indicated by lower HBsAg titers in both cell lysate and culture supernatant (Figure 3A,B and Figure 4A,B). That did not increase the ratio of extracellular/intracellular HBsAg relative to 0.7 mer S construct (Figure 3C and Figure 4C). In this regard the CMV-S construct is highly artificial. It had only the 0.7 kb S gene (genomic positions 155–835) inserted to a mammalian expression vector. The 0.7 mer construct contained, besides the SPII promoter, two HBV enhancer elements and post-transcriptional regulatory element (PRE) for export of unspliced HBV RNAs [25,26], followed by SV40 polyA signal.

The S region harbored 39 divergent nucleotide positions between geno5.4 and geno1.2, leading to 22 amino acid substitutions scattered on the S protein of 226 residues (Figure 7A). Starting with the 0.7 mer S construct of DpreS/AS, 3 chimeric constructs (chi1, chi2, chi4) were generated with part of the S gene of geno5.4 replaced with that of geno1.2 (Figure 7B). In the complementary approach, corresponding chimeras of the 0.7 mer S construct of DpreS/DS were prepared. Transfection experiments in Huh7 cells suggested that the determinant for higher HBsAg secretion by geno1.2 was located in the 3′ 2/3rd of the S region defined by chi2, although part of that sequence (defined by chi4) rather reduced HBsAg secretion (Figure 8). Using shorter chimeric constructs (chi3.1, chi3.2, chi3.3, chi4.1, chi4.2), we could refine the determinant for higher HBsAg secretion of geno1.2 to smaller regions defined by chi3.1 and chi3.2, and the determinant for reduced HBsAg secretion to a smaller region covered by chi4.1 (Figure 9). Site-directed mutagenesis based on both DpreS/AS and DpreS/DS suggested that V118 and T127 in the S protein of geno1.2 promoted HBsAg secretion relative to T118 and P127 present in geno5.4 (Figure 10). Introducing Y161F and V168A substitutions into DpreS/AS further reduced HBsAg secretion, especially in combination (Figure 11), although the reverse mutagenesis on DpreS/DS was not performed. GenBank search suggested that V118 is present only in some subtype D2 isolates. T127 is found in HBV isolates belonging to ayw3 serotype including D2, D3, and C4 subtypes, because it is the determinant for “w3” specificity [27]. On the other hand, all subtypes of genotype D have F161/A168 in contrast to Y161/V168 shared by genotype A clones. V168 is also found in genotypes B and C.

Residues 118, 127, 161, and 168 are all located inside the major hydrophilic region (residues 99–169) of the S protein (exposed on particle surface), although only P127 lies inside the “a” determinant (residues 124–147), the major target of neutralizing antibodies. Interestingly, both T118K and P127S are immune escape mutations that we previously found to severely impair virion secretion when introduced to a genotype A clone (the impact on HBsAg secretion was not determined) [28]. The P127S mutation can abolish the binding of a monoclonal anti-S antibody confirming its immune escape [29]. As for the nearby residues, G119E and G119R are also immune escape mutations that impair both virion and HBsAg secretion [30,31], while S117T was associated with low HBsAg titer in genotype C infected serum samples [32]. I126S mutation in genotype C and T126N mutation in genotype A also impaired virion secretion, with the I126S mutation also known to reduce HBsAg secretion [28,31]. We also found I162T in genotype C can reduce virion secretion [33]. As for A168 in genotype D vs. V168 in genotypes A–C, we previously found nearby R169P mutation in genotype A completely abolished both virion and HBsAg secretion [30]. Moreover, a recent study found V168A mutation in genotype B patients of occult HBV infection [34]. Site-directed mutagenesis revealed ability of this mutation to reduce HBsAg secretion from a genotype B clone without much effect on intracellular level [34]. Therefore, the V168A mutation could reduce HBsAg secretion in the context of genotype A (our current study) and genotype B [34]. Together, these data suggest that the same substitution can lead to genotypic differences in HBsAg secretion and immune escape (A168 in genotype D vs. V168 in other genotypes, and V168A mutation in genotype B), or that different substitutions in the same position lead to genotypic differences vs. immune escape (V118 vs. T118; T127 vs. P127; T118A, T118K, and P127S escape mutations).

In conclusion, the present work confirmed weaker SPII promoter of a genotype D clone leading to reduced HBsAg production. It also confirmed more efficient HBsAg secretion from the S protein of a genotype D clone but a stronger inhibitory effect of its L protein. The current study revealed that the efficiency of HBsAg secretion correlates inversely with the dose of plasmid DNA transfected and consequently amount of S protein expressed, which can partly explain more efficient HBsAg secretion by genotype D than genotype A. Subtype D2 specific residue V118 and subtype D2, D3, and C4 specific T127 in the S protein could promote HBsAg secretion, whereas genotype D specific F161/A168 rather reduced HBsAg secretion. It will be of interest to study the combined effect of V118/T127 in genotype A, to investigate the impact of F161Y/A168V substitutions in genotype D, and to determine the impact of divergent residues 118, 127, 161, and 168 on HBsAg secretion in the context of intact HBV genome (SphI dimer). Finally, whether such substitutions alter the secretion of virions in addition to SVPs warrants further investigation.

## Supplementary Materials

The following are available online at https://www.mdpi.com/1999-4915/12/9/967/s1, Figure S1: Three pairs of chimeric constructs between ApreS/AS and ApreS/DS to narrow down the determinants for efficient HBsAg secretion, Figure S2: Five pairs of shorter chimeric constructs between ApreS/AS and ApreS/DS to further map the determinants for efficient HBsAg secretion.
