# Supplementary material for: Expression Level of Small Envelope Protein in Addition to Sequence Divergence inside Its Major Hydrophilic Region Contributes to More Efficient Surface Antigen Secretion by Hepatitis B Virus Subgenotype D2 than Subgenotype A2
Source: Viruses. 2020 Sep 1;12(9):967. doi: 10.3390/v12090967 (PMC7552069; doi:10.3390/v12090967)
Supplement: Supplementary file 1 [file viruses-12-00967-s001.pdf]

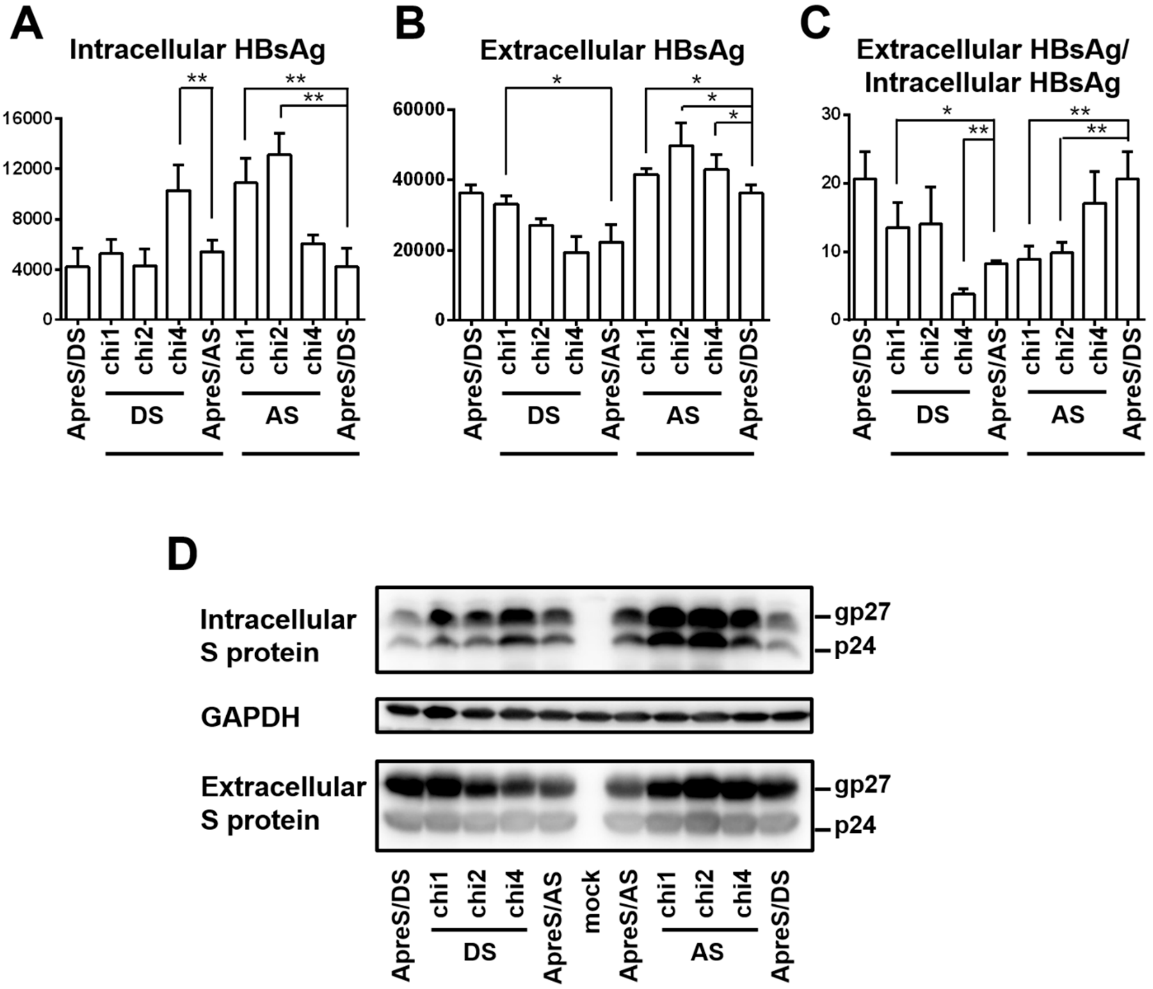

Figure S1. Three pairs of chimeric constructs between ApreS/AS and ApreS/DS to map the determinants for efficient HBsAg secretion. Huh7 cells in six-well plates were transfected with 0.7mer S construct of ApreS/AS, ApreS/DS, or three chimeric constructs, followed by HBsAg measurement from both cell lysate and culture supernatant. For the left part of panels A-D, chi1, chi2, and chi4 with DS are ApreS/AS (lanes 2-4) with various parts of the S region replaced with geno1.2, while ApreS/DS (lane 1) had the entire S region replaced with geno1.2. For the right part, chi1, chi2, and chi4 with AS (lanes 6-8) are ApreS/DS with various parts of the S region replaced with geno5.4, while ApreS/AS (lane 5) had the entire S region replaced. (A-C) Total intracellular (A) and extracellular (B) levels of HBsAg (OD_{450}) averaged from three independent transfection experiments, and the calculated ratio of extracellular/intracellular HBsAg (C). Statistical difference between each chimeric construct and its parental construct was analyzed by One-way ANOVA (*$p<0.05$, **$p<0.01$). (D) Western blot analysis of intracellular and extracellular S protein from same volume of cell lysate or culture supernatant, with GAPDH serving as loading control for cell lysate.

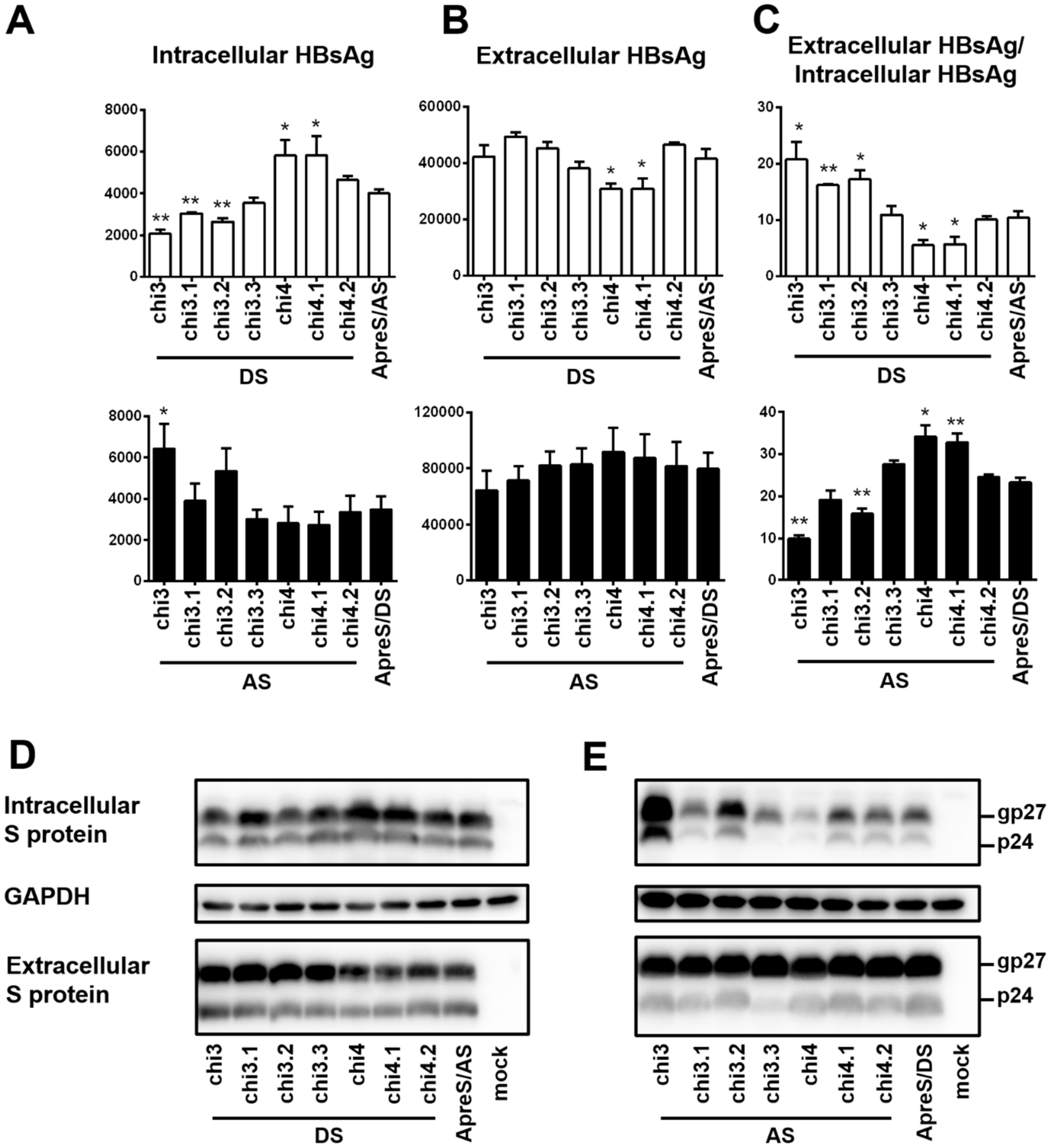

Figure S2. Five pairs of shorter chimeric constructs between ApreS/AS and ApreS/DS to further map the determinants for efficient HBsAg secretion. (A-C) Total intracellular (A) and extracellular (B) levels of HBsAg (OD_{450}) averaged from three independent transfection experiments and the calculated ratio of extracellular HBsAg/intracellular HBsAg (C). The top panels are original ApreS/AS and 7 chimeric constructs with the S region of geno1.2 (DS). Of these, chi3.1, chi3.2, and chi3.3 covered different parts of chi3, while chi4.1 and chi4.2 covered different parts of chi4. The lower panels are ApreS/DS and 7 chimeric constructs with the S region of geno5.4 (AS). Statistical difference between the original construct (ApreS/AS or ApreS/DS) and a chimeric construct was analyzed by One-way ANOVA (*p<0.05, **p<0.01). (D & E) Western blot analysis of intracellular and extracellular S protein from one transfection experiment from the ApreS/AS series (D) and ApreS/DS series (E). GAPDH served as loading control for intracellular S protein.
